# Impaired emotional autobiographical memory associated with right amygdalar-hippocampal atrophy in Alzheimer’s disease patients

**DOI:** 10.3389/fnagi.2015.00021

**Published:** 2015-03-16

**Authors:** Nathalie Philippi, Anne Botzung, Vincent Noblet, François Rousseau, Olivier Després, Benjamin Cretin, Stéphane Kremer, Frédéric Blanc, Liliann Manning

**Affiliations:** ^1^CMRR, Service of Neurology, Neuropsychology Unit, University Hospital of StrasbourgStrasbourg, France; ^2^Cognitive Neuropsychology and Pathophysiology of Schizophrenia (U1114), University of Strasbourg and INSERMStrasbourg, France; ^3^ICube Laboratory (UMR 7357), University of Strasbourg and CNRSStrasbourg, France; ^4^Cognitive and Adaptive Neurosciences Laboratory (UMR 7364), University of Strasbourg and CNRSStrasbourg, France; ^5^Service of Radiology, University Hospital of StrasbourgStrasbourg, France

**Keywords:** emotion, autobiographical memory, Alzheimer’s disease, amygdala, hippocampus

## Abstract

We studied the influence of emotions on autobiographical memory (AbM) in patients with Alzheimer’s disease (AD), characteristically triggering atrophy in the hippocampus and the amygdala, two crucial structures sustaining memory and emotional processing. Our first aim was to analyze the influence of emotion on AbM in AD patients, on both the proportion and the specificity of emotional memories. Additionally, we sought to determine the relationship of emotional AbM to amygdalar-hippocampal volumes. Eighteen prodromal to mild AD patients and 18 age-matched healthy controls were included. We obtained 30 autobiographical memories per participant using the modified Crovitz test (MCT). Analyses were performed on global scores, rates and specificity scores of the emotional vs. neutral categories of memories. Amygdalar-hippocampal volumes were extracted from 3D T1-weighted MRI scans and tested for correlations with behavioral data. Overall, AD patients displayed a deficit in emotional AbMs as they elicited less emotional memories than the controls, however, the specificity of those memories was preserved. The deficit likely implied retrieval or storage as it was extended in time and without reminiscence bump effect. Global scores and rates of emotional memories, but not the specificity scores, were correlated to right amygdalar and hippocampal volumes, indicating that atrophy in these structures has a central role in the deficit observed. Conversely, emotional memories were more specific than neutral memories in both groups, reflecting an enhancement effect of emotion that could be supported by other brain regions that are spared during the early stages of the disease.

## Introduction

Emotions have been widely demonstrated to enhance anterograde memory (e.g., Heuer and Reisberg, 1990; Cahill and McGaugh, 1995) and are additionally closely linked to autobiographical memory (AbM), which allows a person to remember the events of their life in the initial temporo-spatial context and is crucial for building up one’s identity (Conway and Pleydell-Pearce, 2000). The existence of an emotional enhancement of memory for personal events has been shown in healthy young (see Holland and Kensinger, 2010, for a review) and older subjects (Comblain et al., 2005; Schaefer and Philippot, 2005; St Jacques and Levine, 2007).

While a series of studies in Alzheimer’s disease (AD) patients found a preserved effect of emotional enhancement in anterograde memory (Kazui et al., 2000; Moayeri et al., 2000; Boller et al., 2002; Satler et al., 2007), others indicated either the opposite results (e.g., Hamann et al., 2000; Abrisqueta-Gomez et al., 2002; Kensinger et al., 2002, 2004), or a distinct emotional effect for visual and verbal material (Kazui et al., 2003). In a study examining AD patients’ emotional reaction to a shared traumatic event, namely the 1995 Kobe earthquake, Ikeda et al. (1998) showed a reinforcement of memory retention for this intense emotional experience compared with neutral events that occurred during the same period of life. In cases such as those involving anterograde memory, it is difficult to disentangle the phases of memory storage or retrieval vs. memory formation, since all these processes are engaged while the brain is damaged. Conversely, autobiographical memories (AbMs) provide the opportunity to study the influence of emotion on memory retrieval, because this phase is separated from memory formation with a significant time interval. Moreover, memory formation can be considered as being intact for memories that occurred before the onset of a brain lesion. Hence, the study of AbMs is a pertinent method for investigating the influence of emotional states and the consequence of brain lesions on the storage or the retrieval of emotional memories. While AbM has been extensively explored in AD and shown to be deficient, only two studies have mentioned the emotional component of memories, and both concluded that there were no specific effects (Addis et al., 2009; Irish et al., 2011).

While the hippocampus plays a central role in the retrieval of AbMs (review in Svoboda et al., 2006), the enhancement of memory with emotions is also thought to rely on other limbic regions, namely the amygdala. Importantly, the amygdala is involved during the phases of encoding and consolidation (Cahill et al., 1996; Adolphs et al., 1997; Canli et al., 2000; Dolcos et al., 2004; see McGaugh, 2004 for a review), and the phase of retrieval for both laboratory-based stimuli (Dolan et al., 2000; Sharot et al., 2004; Dolcos et al., 2005; Smith et al., 2006) and autobiographical recollections (see Buchanan et al., 2005, 2006; Greenberg et al., 2005; Buchanan, 2007 for a review). The interaction between amygdala and hippocampus was demonstrated in animal experimentation of conditioned learning during encoding, consolidation (Packard and Cahill, 2001) and retrieval phases (e.g., Seidenbecher et al., 2003). Interestingly, such conditioned learning is deficient in AD mice models due to altered long-term potentiation within hippocampal synapses (e.g., Dominguez-del-Toro et al., 2004; Gruart et al., 2008). Despite the fact that the amygdala and other limbic structures related to emotional and memory processing are damaged in the early stages of AD (Hopper and Vogel, 1976; Braak and Braak, 1991), little is known about emotional AbM in these patients and its relationship with limbic structure volume.

The aim of the present study was to analyze the influence of emotion on AbM in AD patients compared with elderly controls, both *quantitatively* (using the proportion of the retrieved emotional memories) and *qualitatively* (in measuring the degree of specificity). We also analyzed the pattern of performance over the lifespan to determine if the potential deficit of emotional memory would be time-extended and related to a storage/retrieval deficit, or restricted to the most recent periods of life and related to a deficit of memory formation. Additionally, we sought to investigate the relationship between a potential deficit in emotional AbM and the degree of atrophy in the hippocampus and amygdala. Given the central role of the amygdala in emotional memory enhancement and since this structure is damaged in the early stages of AD, we predicted that a deficit of emotional AbM would exist in AD patients in comparison to normal elderly participants. We also hypothesized that emotional AbM deficits would be related to the degree of atrophy in the amygdala.

## Materials and Methods

### Participants

Eighteen French AD patients, aged 67–85 years old, were recruited through the Neuropsychology Unit in the Service of Neurology at the University Hospital of Strasbourg. A diagnosis of probable AD was made according to the criteria of the NINCDS-ADRDA (McKhann et al., 1984) and to more recent criteria (Dubois et al., 2007; Albert et al., 2011; McKhann et al., 2011). Nine patients had mild cognitive impairment (MCI) due to AD or prodromal AD, according to the criteria proposed by Albert et al. (2011) and Dubois et al. (2007), which include preserved social functioning and instrumental activities of daily living. All of them evolve into a typical dementia due to AD with amnestic presentation within one or two years during the follow-up. The 9 remaining patients had mild dementia. Each patient disclosed a history of progressive cognitive decline and showed objective impairment of episodic memory with no cueing-related improvement (Free and Cued Selective Reminding Test Grober et al., 1988), either isolated or associated with other cognitive changes such as visuospatial abilities, praxis or language. Evidence of MTL atrophy (Barkhof et al., 2007) was found in all patients on MRI images, visually examined by a senior neurologist (FB). A subset of twelve patients underwent SPECT examination (including six of the prodromal patients), which revealed a pattern of reduced perfusion in the MTL and parietal regions. Eleven patients underwent a cerebrospinal fluid (CSF) biomarker testing (including the 9 prodromal patients). The results were abnormal, showing a combination of low amyloïd Abeta1–42 concentrations (<500 ng/L), increased total tau concentrations (>500 ng/L) and increased phospho-tau concentrations (>60 ng/L). We excluded patients who disclosed a history or symptoms of major depression (Geriatric Depression Scale’s score >6, Sheikh and Yesavage, 1986), cerebro-vascular disease, abnormal physical neurological examination, or any other condition potentially leading to dementia. All patients taking one or two AD specific drugs (acetylcholine esterase inhibitor or acetylcholine esterase inhibitor and memantine). The 9 patients with prodromal AD had preserved social functioning and instrumental activities of daily living with Clinical Dementia Rating score of 0.5 (CDR; Hughes et al., 1982), while the 9 patients with mild dementia had a CDR score of 1. The patients’ mean score on the MMSE was 24.3 (SD 2.8). In addition to the AbM test, the patients’ anterograde memory was also assessed using the Verbal Paired Associates (Wechsler, 1991), in which the patients obtained a mean score of 9.0 (SD 4.2). A subset of 15 patients underwent a high resolution MRI scan, within 6 months from neuropsychological testing.

Eighteen healthy elderly subjects matched for gender, age distribution, education level and handedness (see Table 1) were also tested. Inclusion criteria comprised a normal neurological examination, and absence of depression (GDS score >6), central neurological disease, cognitive complaint or restriction of daily activities (CDR = 0). The control subjects also underwent both the AbM test and the Verbal Paired Associates test (Wechsler, 1991). They obtained normal scores on this latter test (mean 18.0 and SD 1.7).

**Table 1:** **Demographic data for the AD patients and the controls**.

	*N*	Sex ratio (F/M)	Mean age (SD) in years	Mean education level (SD) in years	Handedness (L/R)
Control group	18	4/14	73.67 (5.37)	13.17 (3.01)	1/17
Patient group	18	4/14	77.17 (6.42)	12.17 (6.68)	1/17
Statistical analysis	-	-	*t*_(34)_ = 1.17 *p* = 0.08	*t*_(34)_ = −1.05 *p* = 0.3	-

*F: female; M: male; L: left; R: right; SD: standard deviation*.

All participants provided informed written consent for the study according to the Declaration of Helsinki and the MRI procedure was approved by the local Ethics committee.

### Autobiographical Memory Task

A French version (Manning, 2002) of the modified Crovitz Test (MCT, Graham and Hodges, 1997) was used to assess AbM. Participants were asked to produce detailed and specific recollections in response to 6 specific cues (e.g., letter, train, surprise…), each prompted five times in order to elicit memories from five life periods (“0–9 years”, “10–29 years”, “30–59 years”, 60 to current age minus 1 year—referred as “after 60 years”-, and “previous year”; e.g., “Could you recall an event that happened in relation to *a train* before* you turned 9*”). Presentation of the words and time periods were randomized and no time-limit was set. A second cue was given for each possible response before considering that a participant had no memory. The participants were encouraged to recall as many details as possible (spatial, temporal, and emotional) and incomplete responses were probed with further questions (e.g., “Tell me more.”; “Do you remember where it took place?”). Memories were subsequently scored on a 5-point specificity scale, and were divided into five different categories: 0, absence of response; 1, semantic facts related to the target word; 2, poorly detailed generic or repeated events; 3, detailed generic or repeated events; 4, poorly detailed specific events; 5, richly detailed specific events. Only memories for events associated with a unique spatial and temporal context were considered as episodic (“specific”) and scored 4 or 5 depending on their level of detail, while other memories were considered as “general”. Thirty memories per participant were thus prompted for recollection, with a maximum possible score of 150. Half of these memories were scored by two different raters (NP and LM) with no significant inter-rater differences. Independently of the level of details and the episodic/semantic nature of the recollections, for each memory, participants were asked to state whether they were emotional at the time of living the event. For all patients except two, a relative was present during the autobiographical assessment, allowing the examiner to check for accuracy of the memories.

### Hippocampal and Amygdalar Volumetry

A subgroup of 15 patients underwent a high resolution MRI scan within 6 months of neuropsychological testing. High resolution anatomical images were obtained with a General Electric SIGNA HDx MR 3T MRI (Milwaukee, USA) using a Fast Spoiled Gradient Echo sequence (TR = 7.2 ms, TE = 2.3 ms, flip angle = 20°, FOV = 22 cm, matrix = 256 × 256, 176 slices of 1 mm). Hippocampal and amygdalar volumes, as well as the total gray matter volume, were investigated using the subcortical labeling method (Fischl et al., 2002) provided in the freesurfer software.^1^ Five steps were followed: an affine registration with Talairach space specifically designed to be insensitive to pathology and to maximize the accuracy of the final segmentation; then, an initial volumetric labeling precedes correcting for intensity variation due to the B1 bias field inhomogeneity. The forth step consists in performing a high dimensional nonlinear volumetric alignment to the Talairach atlas. Finally, the volume is labeled based on a subject-independent probabilistic atlas that is built from a training set of subjects mapped into the Talairach space whose brains have been labeled by hand.

### Statistical Analysis

#### Behavioral Data

Inter-group comparisons were tested using Student’s *t*-test for quantitative demographic characteristics (age distribution and education level). Two different categories of autobiographical memories were defined as “neutral” and “emotional”, independently of the level of detail (scored 2 to 5). Reference to “scores” (“emotional score”, “neutral score”) entails both the number of retrieved memories and their level of detail (up to 30 [maximum number of retrieved memories] *5 [specificity-score] = 150 maximum score). To clearly distinguish the effect of emotion on both *quantitative* and *qualitative* aspects of recollection, we also explored the results in terms of (i) the “rate” of memories in each category relative to the total number of retrieved memories (%) and (ii) the memories’ “specificity-score” on the MCT’s 5-point scale as a reflection of the level of episodic details for each category. Additionally, we studied the effect of the periods of life on “emotional scores” compared with “neutral scores”. ANCOVAs were performed to test potential interactions between the groups and the categories of memories for the scores, rates, and specificity scores, including age as a covariate. Moreover, we used ANCOVAs to test potential interactions between the periods and the categories of memories per group for the scores on the MCT, including age as covariate. Finally, we also performed ANCOVAs to test potential interactions between the periods and the groups depending on the category of memories, including age as a covariate. *Post hoc* analyses were performed using Tukey’s test. In order to control for possible violations of the homogeneity of variance associated with analysis of binary data summarized as percentages, an arcsine transformation was performed on the rates of memories and statistical tests were conducted using the transformed data. After transformation of data, normality of the distribution was verified using the Kolmogorov-Smirnov test.

#### Volumetric Data

We tested the existence of partial correlations between amygdalar-hippocampal volumes (left and right hippocampus, left and right amygdala) and behavioral data (emotional as compared with the neutral scores), after exclusion of age effect and total gray matter volume. We choose to introduce total gray matter as nuisance covariate because this allows to take into account both the initial brain volume and the global effect of atrophy due to AD and aging. Thus, the potential correlations obtained with the amygdalar-hippocampal volume would not be the result of the global disease progression (Peelle et al., 2012). Positive correlations were expected between the emotional scores and the volume of the amygdala. Bonferroni correction for multiple analyses was applied using a statistical threshold of *p* = 0.006 (0.05 divided by the number of categories (*n* = 2) and by the number of brain structures tested (*n* = 4)). When positive correlations were obtained, we tested whether they were due to the rate of the elicited memories, to their level of detail, or both. Additionally, to determine whether the amygdalar-hippocampal structures were involved independently of the retention interval, we analyzed the relationships between the volumes of these structures and the emotional scores for the five life periods. Bonferroni correction was also applied for this additional independent question, using a threshold of *p* = 0.01 (i.e., 0.05 divided by the number of periods).

## Results

### Emotional vs. Neutral Memories

ANCOVAs showed a significant group effect (*F*_(1,33)_ = 32.6; *p* < 0.001), no significant category effect (*F*_(1,33)_ = 0.14; *p* = 0.71), and a significant group—category interaction (*F*_(1,33)_ = 10.6; *p* = 0.002) for the mean scores. Regarding the mean rates (arsine transformation) of retrieved memories, there was no significant group effect (*F*_(1,33)_ = 0.47; *p* = 0.50), no significant category effect (*F*_(1,33)_ = 0.27; *p* = 0.61), but a significant group—category interaction (*F*_(1,33)_ = 11.8; *p* = 0.001). Finally, ANCOVAs showed a significant group effect (*F*_(1,31)_ = 16.7; *p* < 0.001), but no significant category effect (*F*_(1,31)_ = 0.04; *p* = 0.83) and no significant group—category interaction (*F*_(1,31)_ = 2.75; *p* = 0.11) for the mean specificity scores (See Table 2).

**Table 2:** **Comparison of different scores (/150), rates of memories relatively to the total number of memories (%) and specificity-scores (/5-point scale) in the emotional and neutral categories of memories on the MCT between AD patients and controls**.

MCT scores	Category of the memory	AD patients	Controls	Inter-group comparisons
Mean scores (/150)	Emotional	34.9 (SD 28.4)	77.3 (SD 17.0)	p < 0.001**
	Neutral	39.0 (SD 15.6)	41.2 (SD 13.3)	*p* = 0.99
Mean rates of memories (%)	Emotional	35.1 (SD 23.1)	60.9 (SD 11.6)	p = 0.002*
	Neutral	64.9 (SD 23.1)	39.1 (SD 11.6)	p = 0.002*
Mean specificity-scores (/5)	Emotional	3.9 (SD 0.5)	4.3 (SD 0.3)	*p* = 0.08
	Neutral	2.7 (SD 0.6)	3.6 (SD 0.3)	p < 0.001**

SD: standard deviation, bold and **indicate a significant difference with *p* < 0.001). Statistical analyses were performed using Tukey’s test.

#### Inter-Group Comparisons

*Post hoc* analyses within the emotional category of memories, showed that the mean scores and the mean rates (arcsine transformation) of retrieved memories were significantly lower for AD patients vs. controls (*p* < 0.001 and *p* = 0.002, respectively), but it was not the case for the mean specificity-scores for emotional memories (*p* = 0.08). With regards to neutral memories, the mean scores were not significantly different between the groups (*p* = 0.99). Indeed, AD patients had higher mean rates of neutral memories than controls (*p* = 0.002) but lower mean specificity-scores for this category (*p* < 0.001).

#### Intra-Group Comparisons

***In the control group***, *post-hoc* analyses showed that the mean scores (*p* < 0.001) and the mean specificity-scores (*p* < 0.001) of the retrieved memories were significantly higher in the emotional category than in the neutral category, but it was not the case for the mean rates (arsine transformation; *p* = 0.19). ***In the patient group***, *post-hoc* analyses showed that the mean scores of neutral and emotional memories were similar (*p* = 0.95), with higher mean rates (arcsine transformation) of neutral memories (*p* = 0.007) but higher mean specificity-scores of emotional memories (*p* < 0.001).

### Effect of Time Period on Emotional vs. Neutral Memories

**Figure 1:**
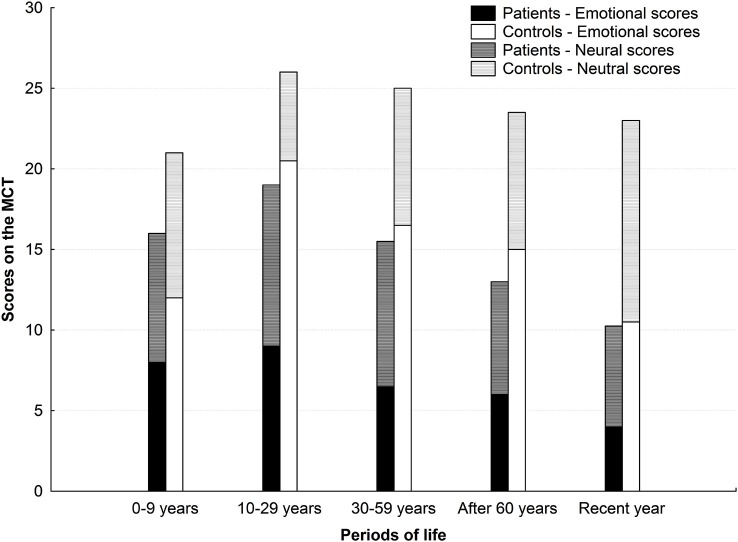
**Mean AbM scores on the MCT for the five periods of life in AD patients (black) and controls (white)**. The mean scores entail both the number of retrieved memories (up to 6 per period) and their level of detail (specificity score on a five-point scale), with a maximum score of 30 per period. The emotional scores and neutral scores are summed and indicated in black and dark gray for the AD patients, and in white and light gray for the controls, respectively.

The mean scores on the MCT, including the summed emotional and neutral scores, are shown in Figure 1 as a function of the periods of life in both groups.

#### Period-Category Interaction Depending on the Group

***In the control group***, ANCOVAs showed a significant category effect but no period effect for the mean emotional scores (*F*_(1,33)_ = 49.1; *p* < 0.001 and *F*_(4,132)_ = 0.23; *p* = 0.92, respectively). A significant interaction between period and category was shown for this group (*F*_(4,132)_ = 11.3; *p* < 0.001). *Post hoc* analyses revealed that the healthy control group had higher emotional than neutral scores for the following life periods “10–29 years”, “30–59 years” (*p* < 0.001 in both cases), and “after 60 years” (*p* = 0.006). Within the emotional category of memories, scores were higher for the “10–29 years” period compared with the “0–9 years” (*p* < 0.001), “after 60 years” periods (*p* = 0.02), and “recent year” (*p* < 0.001). Emotional scores from the “recent year” were also lower than in the “30–59 years” period (*p* = 0.009). Within the neutral category of memories in the control group, no significant differences were shown between the different periods.

***In the patient group***, no significant category effect (*F*_(1,33)_ = 0.30; *p* = 0.59) and no significant period effect (*F*_(4,132)_ = 0.27; *p* = 0.90) were shown; in this group, there was no significant interaction between the period and the category (*F*_(4,132)_ = 1.0; *p* = 0.41).

#### Period-Group Interaction Depending on the Category of Memories

In order to be able to compare scores between the two groups, we also performed ANCOVAs to test potential interaction between periods and groups. A significant group effect and period effect was revealed for the mean emotional scores (*F*_(1,33)_ = 22.3; *p* < 0.001), but no significant period effect (*F*_(4,132)_ = 1.3; *p* = 0.26); no significant group effect or period effect was found for the mean neutral scores (*F*_(1,33)_ = 0.003; *p* = 0.96 and *F*_(4,132)_ = 2.45; *p* = 0.05) ; a significant interaction between the period and the group existed both for the emotional and neutral scores (*F*_(4,132)_ = 3.9; *p* = 0.004; *F*_(4,132)_ = 3.8; *p* = 0.005, respectively). *Post hoc* analyses revealed that the patients’ mean emotional scores were significantly lower than the controls’ for each period of life (*p* < 0.001 for the “10–29 years” and “30–59 years” periods, *p* = 0.006 for the “after 60 years” period, and *p* = 0.03 for the “recent year”) but the “0–9 years” period (*p* = 0.67). For the mean neutral scores, there was no significant difference between the two groups (*p* = 1.0 for the “0–9 years” period, *p* = 0.87 for the “10–29 years” period, *p* = 1.0 for the “30–59 years” period, *p* = 1.0 for the “after 60 years” period, and *p* = 0.95 for the “recent year”).

### Amygdalar-Hippocampal Volumetry

**Figure 2:**
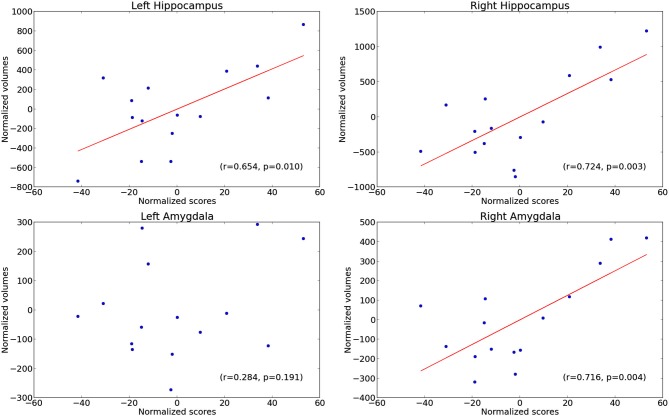
**Scatter plots of the correlation analyses performed between emotional scores and volumes of the amygdala (lower) and hippocampus (upper panels) on the left (panels on the left) and on the right (panels on the right)**.

Figure 2 shows the scatter plots of the data used for the correlation analyses performed in the AD patient group between emotional scores and volumes of the amygdala and the hippocampus.

We found a significant positive correlation between emotional scores on the MCT over the lifespan and the hippocampal volume, bilaterally (right: *p* = 0.003; *r* = 0.72; left: *p* = 0.01; *r* = 0.65), and the volume of the right amygdala (*p* = 0.004; *r* = 0.72) only (left amygdala: *p* = 0.19; *r* = 0.28), whereas no positive correlation were found for neutral scores (*p* = 1 and *r* < 0 in every case). The emotional memory correlations remained significant only on the right hemisphere after correction for multiple comparisons using a threshold of *p* = 0.006, since four different structures and two different categories of memories were tested.

To determine whether amygdalar-hippocampal structures were associated in relation to the number of memories elicited or their level of detail or both, we ran correlation analyses between the volume of these cerebral structures and the rates of emotional memories, in the one hand, and the specificity scores, on the other. Interestingly, correlations were also obtained for the rates of emotional memories (*p* = 0.03 and *r* = 0.54 for the right hippocampus, and *p* = 0.05 and *r* = 0.49 for the right amygdala), but not with the specificity scores of the emotional category (*p* = 0.25 and *r* = 0.25 for the right hippocampus, *p* = 1 and *r* < 0 for the left hippocampus, and *p* = 0.10 and *r* = 0.46 for the right amygdala).

To detect a potential involvement of the amygdalar-hippocampal structures over the lifespan, we carried out correlations independently for each life period. Emotional scores correlated with the right hippocampal volume for all life periods but the first (*p* = 0.02 and *r* = 0.60 for the “10–29 years” period, *p* = 0.0003 and *r* = 0.81 for the “30–59 years” period, *p* = 0.007 and *r* = 0.67 for the “after 60 years” period, *p* = 0.03 and *r* = 0.54 for the “previous year”) and with the left hippocampus for the three intermediate periods (*p* = 0.009 and *r* = 0.66 for the “10–29 years” period, *p* = 0.008 and *r* = 0.67 for the “30–59 years” period, *p* = 0.01 and *r* = 0.64 for the “after 60 years” period). Emotional scores were also correlated with the volume of the right amygdala for all life periods but the first (*p* = 0.02 and *r* = 0.60 for the “10–29 years” period, *p* = 0.001 and *r* = 0.76 for the “30–59 years” period, *p* = 0.01 and *r* = 0.64 for the “after 60 years” period, *p* = 0.02 and *r* = 0.58 for the “previous year”). Only correlations with a threshold of *p* < or = 0.01 remained significant after correction for multiple analyses, considering the five different life periods.

## Discussion

The aim of the present study was to analyze the influence of emotion on AbM in AD patients in comparison with elderly healthy controls, and to study the relationships between deficit in emotional AbM and the degree of hippocampal and amygdalar atrophy. Compared with healthy controls, AD patients showed impaired emotional scores on the MCT, and a lower rate of emotional memories. However, for the emotional memories still accessible for AD patients, the degree of specificity was preserved in comparison with their own neutral memories and the healthy controls’ emotional memories. In other words, AD patients had an overall emotional AbM impairment, which could be explained by a *quantitative* deficit, whereas their remaining memories would be *qualitatively* enhanced with emotion. Finally, we have demonstrated positive correlations between the emotional scores and the volumes of the right amygdala and the hippocampus in AD patients, for the global emotional scores and rates of memories, and no correlation for the specificity scores.

### Emotional Autobiographical Memory During Normal Aging

In line with previous studies of AbM during normal aging (Comblain et al., 2005; Schaefer and Philippot, 2005; St Jacques and Levine, 2007; see Holland and Kensinger, 2010, for a review), we found an enhancement effect of emotion on AbMs in healthy controls. Based on our data, this effect was observed both on the proportion of the retrieved emotional memories and their degree of specificity. While most studies agree that a memory would more likely be accessed when emotionally colored (see Buchanan, 2007, for a review), the role of emotion on the degree of specificity associated with AbMs remains controversial. According to some authors, emotions would increase the level of contextual details (Comblain et al., 2005; St Jacques and Levine, 2007), or it would be only associated with vividness of remote AbMs according to other studies (Schaefer and Philippot, 2005).

We found a differential effect of emotional memory enhancement during normal aging, depending on the life period, with greater scores on the MCT for the “10–29 years” period, revealed by higher emotional scores. This period corresponds to the “reminiscence bump” (Rubin and Schulkind, 1997), which comprises memories from adolescence and young adulthood. These memories are thought to remain more stable over time as they consist of events that are “self-defining”. In accordance with previous studies (Rubin and Berntsen, 2003; Haque and Hasking, 2010), our results seem to confirm the central role of emotions at the time of encoding, which would account for the fact that subjects recall more memories from this period of life, which would also be more detailed (but see Janssen and Murre, 2008 for opposite results).

### Deficient Emotional Autobiographical Memory in AD

Based on the scores on the MCT, we showed an impairment of overall emotional AbMs in AD compared with normal aging, with a time-extended pattern of deficits and without an emotional reminiscence bump effect. Since AD patients were healthy at the time of memory encoding, this time-extensive deficit appears likely to affect either the storage or the retrieval processes. Conversely, a deficit of emotional enhancement uniquely related to memory formation would have been expected for the recent period only (i.e., after the disease onset). It is likely that the patients had an additional deficit of emotional memory formation for the “recent year” period where their scores were lower, however the difference was not significant as compared with the other life periods. A deficit of emotional enhancement has already been demonstrated in relation to memory formation in studies focusing on anterograde memory in AD (Hamann et al., 2000; Abrisqueta-Gomez et al., 2002; Kensinger et al., 2002, 2004; Kazui et al., 2003) and correlated to the degree of atrophy in the amygdala (Ikeda et al., 1998; Mori et al., 1999). Our study complements these previous observations, regarding the storage/retrieval phases, since this is, to our knowledge, the first to highlight a deficit of remote memory for emotional events in AD (no significant difference was found in Addis et al., 2009; Irish et al., 2010). Interestingly, the same pattern of deficits has been shown in studies of patients presenting with MTL lesion (Buchanan et al., 2005, 2006).

Taking into account the rates of accessed memories and their degree of specificity, we were able to attribute this deficit primarily to the fact that AD patients elicited a smaller number of emotional memories. Conversely, the specificity score associated with the accessed emotional AbMs remained preserved in the patients as compared with the controls and in contrast to neutral memories. Consistent with the results obtained in the control subjects (see section Emotional autobiographical memory during normal aging), this finding reflects a reinforcement of episodic details from an enhancement effect of emotional memories. Since this enhancement of the level of details was preserved in the AD patients, one might hypothesize that the reinforcement of the memory traces operated at the level of memory formation, before the onset of AD.

The fact that the emotional component of memories seems linked to their episodic characteristics, could suggest that it is recollected in the same manner as other contextual details (e.g., Levine and Pizarro, 2004), or reconstructed *based on* the contextual details, through the process of episodic remembering (e.g., Robinson and Clore, 2002). However, using the “rates” rather than the number of memories in our analyses, we showed that emotional memory was deficient in the AD group *relative* to neutral AbM, suggesting the existence of independent mechanisms underlying the emotional component and the episodic details of a memory. Furthermore, together with the observations of preserved memory for emotional information despite impaired memory for contextual details (e.g., Tranel and Damasio, 1993), we observed that AD patients elicited some emotional memories lacking contextual details (e.g., with a specificity score of 3). This would additionally favor distinct memory processes for event details and emotional content (see Holland and Kensinger, 2010, for a review). This emotional memory deficit could also result from a diminished re-experiencing of emotion during retrieval due to a general deficit of processing emotional information, which has been observed in AD patients (e.g., Hargrave et al., 2002 for facial recognition of emotions). Though, observations of impaired memory enhancement with emotions and preserved emotion recognition could indicate that memory emotional enhancement exists independently of general emotion processing (e.g., Hamann et al., 2000).

### Neuro-Anatomical Correlates of Emotional Memory Loss in AD

We found positive correlations between AbM loss in AD patients and the degree of atrophy of the right amygdala and hippocampus when considering the emotional category of AbMs, whereas it was not the case with the neutral category. In addition to the well-known implication of the hippocampus in the retrieval of episodic AbM (review in Svoboda et al., 2006), our results confirmed the central role of the two structures in the retrieval of emotional AbMs. The interactions between amygdala and hippocampus were previously demonstrated to mediate emotional arousal of memories in healthy subjects (Cahill et al., 1996; Canli et al., 2000; Dolcos et al., 2004; McGaugh, 2004; review in LaBar and Cabeza, 2006). These correlations were found for all periods of life except childhood, confirming that the amygdala and hippocampus are involved in emotional enhancement of memory for extensive retention intervals, beyond the phase of memory formation. This is consistent with fMRI studies conducted with healthy subjects, which have shown the involvement of the amygdala for the retrieval of both recently learned material (Dolan et al., 2000; Sharot et al., 2004; Dolcos et al., 2005; Smith et al., 2006; Botzung et al., 2010) and remote events (Markowitsch et al., 2000; e.g., Greenberg et al., 2005; see Buchanan, 2007, for a review).

These positive correlations were also observed for the rate of emotional memories, suggesting that the amygdala would increase the likelihood that an emotional experience is recalled, as was the case for the enhanced connectivity between the amygdala and the hippocampus in fMRI studies conducted in healthy subjects (Markowitsch et al., 2000; Sharot et al., 2004; Dolcos et al., 2005; Greenberg et al., 2005; Smith et al., 2006; Daselaar et al., 2008). According to some authors, the activity of the amygdala would also support qualitative aspects of recollection, such as the level of episodic details and/or the feeling of re-experiencing (Markowitsch et al., 2000; Sharot et al., 2004; Kensinger and Schacter, 2005; Smith et al., 2005), though we found no correlation between the amygdalar volume and specificity scores. Indeed, correlational analyses can only reveal associations between impaired behavioral scores and regions of atrophy, and the specificity scores were not impaired in the patients. Our results suggest that other structures might be implied to support the level of details associated with emotional memories. This could be the case for regions that are spared during the initial stages of AD, such as the visual cortices (Markowitsch et al., 2003; Piefke et al., 2003) and/or frontal regions (Piefke et al., 2003; Greenberg et al., 2005; Daselaar et al., 2008). An additional fMRI would help to confirm this hypothesis.

Concerning the hemispheric lateralization, we found a preferential involvement of the right amygdala, which is consistent with previous results: patients with MTL damage in the right hemisphere including the amygdala produce AbMs that are impoverished in their emotional content (Cimino et al., 1991; Buchanan et al., 2006). This hemispheric laterality for emotional processing was also found during intracarotid amobarbital procedures (Ahern et al., 1991; Ross et al., 1994) and was confirmed by neuroimaging studies (Fink et al., 1996; Markowitsch et al., 2000; Demaree et al., 2005).

## Conclusion

Our study has several limitations, such as the absence of measurement of AbMs’ emotional arousal or valence, the evaluation of emotional states at the time of retrieval and of the nature of the elicited episodic details (e.g., visual, sensorial, etc). However, this study is the first to demonstrate a deficit of emotional retrograde memory in AD patients and its association with amygdalar-hippocampal atrophy. Thus, AD patients, as compared with healthy elderly participants elicited less emotional AbMs, though characterized by a greater specificity than neutral memories. This would favor the occurrence of emotional AbM deficits in terms of the *number* of retrieved recollections. On the other hand, there is the residual enhancement effect of emotions on the *qualitative* aspects of emotional memories, similar to that found in healthy controls. Finally, we demonstrated that the emotional memory deficit was correlated with the atrophy of the right amygdala and hippocampus, confirming the crucial role of these structures in the retrieval of remote emotional memories. Overall, a better understanding of the deficit of emotional AbM might help developing AbM therapy programs using emotional material and aimed at supporting the patients’ self and identity (e.g., Irish et al., 2006; review in Caddell and Clare, 2011).

## Conflict of Interest Statement

The authors declare that the research was conducted in the absence of any commercial or financial relationships that could be construed as a potential conflict of interest.

## Footnotes

^1^http://surfer.nmr.mgh.harvard.edu/
